# Modification of biochar by phosphoric acid *via* wet pyrolysis and using it for adsorption of methylene blue

**DOI:** 10.1039/d3ra00680h

**Published:** 2023-05-22

**Authors:** Jia Xu, Meiyuan Fu, Qianhui Ma, Xiaopeng Zhang, Chenghang You, Zaifeng Shi, Qiang Lin, Xianghui Wang, Wen Feng

**Affiliations:** a Key Laboratory of Water Pollution Treatment and Resource Rouse of Hainan Province, Key Laboratory of Soil Pollution Remediation and Resource Utilization of Haikou, College of Chemistry and Chemical Engineering, Hainan Normal University Haikou 571158 China; b State Key Laboratory of Marine Resource Utilization in South China Sea, Hainan Provincial Key Lab of Fine Chemistry, School of Chemical Engineering and Technology, Hainan University Haikou 570228 China

## Abstract

Algae biochar (ABC), coconut shell biochar (CSBC), and coconut coat biochar (CCBC) were prepared by wet pyrolysis in a phosphoric acid solvent under normal pressure. Materials were characterized for their micromorphology, specific surface area, and surface functional groups by scanning electron microscopy (SEM), Brunauer–Emmett–Teller (BET) nitrogen adsorption–desorption spectrum technique and Fourier transform infrared diffraction (FT-IR). The evaluation of the liquid-phase adsorption performance using methylene blue (MB) as a pigment model, and the effects of temperature, pH, adsorbent dosage, and pollutant concentration of the MB adsorption onto modified biochars were fully investigated. The adsorption mechanism was proposed based on the adsorption kinetics curve and adsorption isotherm. The synthetic biochar showed great adsorption properties toward cationic dyes rather than anionic dyes. Specifically, the adsorption abilities for algal biochar, coconut shell biochar, and coconut coat biochar were determined to be 97.5%, 95.4% and 21.2%, respectively. The isothermal adsorption of MB by the three kinds of biochar conformed to the Langmuir equation, and the adsorption process fitted to the quasi-second-order kinetic equation, which suggested that ABC and CSBC effectively adsorbed MB dye molecules through hydrogen bonding, π–π stacking, and electrostatic interactions.

## Abstract 2

Algae biochar (ABC), coconut shell biochar (CSBC), and coconut coat biochar (CCBC) were prepared by wet pyrolysis in a phosphoric acid solvent under normal pressure.

## Graphical abstract

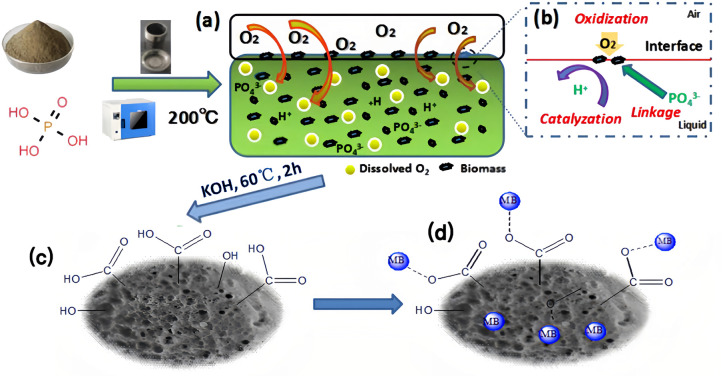

## 1. Introduction

The treatment of dye wastewater has always been an environmental problem that has attracted people's attention.^1^ Most of the industrial wastewater in China's manufacturing industry is composed of many toxic dyes,^2^ which are harmful to the environment and aquatic organisms. At present, the removal methods of dye pollutants in water mainly include the ion exchange method,^3^ oxidation method,^4^ biodegradation method,^5^ adsorption method,^6^*etc.* Adsorption is a method to remove dye pollutants by physical or chemical adsorption with an adsorbent, which is simple to operate, low in cost, and high in efficiency.

Methylene blue (MB) is a common aromatic heterocyclic cationic dye in wastewater, which has low toxicity and significant harm to health after long-term contact.^7^ Therefore, how to remove MB pollutants efficiently has become a concern. In recent years, the adsorbents for removing methylene blue mainly include magnetic nanometer particles,^8^ activated carbon,^9^ and biomass carbon.^10^ Comparatively speaking, biochar prepared from renewable biomass is more in line with the development trend of green chemistry, and is a critical way to improve environmental pollution and achieve efficient use of energy.

Biochar is usually prepared by pyrolysis at high temperatures and without oxygen.^11^ However, this method usually suffers from the problems of both the multiple complex treatment processes and the high energy input, which makes it a costly and complex process, and then affects the large-scale commercialization of biochar adsorbent. Therefore, it is necessary to explore a simple and low-power preparation method for biochar. In contrast, the wet pyrolysis can occur the reaction in an aqueous medium at a relatively low temperature.^12^ As compared to the pyrolysis at high temperature, this wet process can effectively reduce the energy demand of the reaction condition. Moreover, various functional biochar can be formed in one step reaction through the direct addition of active reagents into reaction solution during the carbonization and activation processes.

In fact, the surface-functionalized biochar can be obtained *via* both simultaneous conversion and activation.^13,14^ During biochar formation, a liquid/gas interface is generated, allowing oxygen to dissolve and causing the surface of biochar to be oxidized. Thus generating enough functional groups on the surface can enhance the adsorption performance. In this experiment, seaweed, coconut shell, and coconut coat were selected as biomass materials to prepare biochar by wet pyrolysis method using phosphoric acid as solvent. The adsorption performances of methylene blue on biochars were studied.

## 2. Materials and methods

### 2.1 Materials

The seaweed was collected from Guilin Ocean Beach, Haikou. The coconut shell and coconut coat were prepared by separating edible coconut. The phosphoric acid and potassium hydroxide were supplied from Sinopharm Chemical Reagent Co., Ltd. (Shanghai, China). Methylene blue was supplied from Shanghai McLean Biochemical Technology Co., Ltd. (Shanghai, China). Hydrochloric acid was supplied from Xilong Science Company (Shanghai, China).

### 2.2 Preparation and characterization of biochars using wet-pyrolysis system

Initially, the raw seaweed was cleanly washed using distilled water. Then these seaweed materials were dried at 70 °C to remove all free water. The dehydrated materials were crushed and screened with 100 mesh. Subsequently, a seaweed suspension mixture was prepared by adding seaweed powder (1.6 g) into 20 mL of 75 wt% phosphoric acids. After stirring over 2 h, the resulting mixture was transferred into a 100 mL steel autoclave with a sealing cap, which was further heated at 200 °C for 5 h. After the autoclave was naturally cooled to the room temperature, the resulting raw biochars were collected through centrifugation. Then a mixture of as-prepared biochar with 20 mL of potassium hydroxide (KOH, 2 mol L^−1^) was prepared and kept at 70 °C for 6 h. The resulting powders were centrifuged, and then washed with distilled water until neutral. After being dried at 70 °C under a vacuum oven for 12 h, the desired adsorbent algae biochar (ABC) sample was obtained. The other two biochars coconut shell biochar (CSBC), and coconut coat biochar (CCBC) were prepared from coconut shells and coconut coats by the same method.

Field emission scanning electron microscopy and energy dispersion spectroscopy were used to analyze the microscopic morphology (SEM-EDS); the surface functional groups were analyzed by Fourier transform infrared spectroscopy (FT-IR), KBr tableting, measuring range 400–4000 cm^−1^; according to Brunauer–Emmett–Teller (BET) equation, the specific surface area was calculated by surface area analyzer based on nitrogen adsorption/desorption records at 77 K; the absorbance of methylene blue solution was measured by UV-visible spectrophotometer at the maximum absorption wavelength of 662 nm, and the removal rate and adsorption capacity were calculated. The zero point potential of the biochar surface was measured concerning the literature method.^15^

### 2.3 Adsorption of methylene blue on biochars

The methylene blue (MB) dye was employed as a pollutant for adsorption performance test in this work. The corresponding adsorption ability of modified biochar prepared by hydrothermal phosphoric acid in dye wastewater was investigated. Typically, 10 mg of modified biochar and 25 mL of MB solution (100 mg L^−1^, pH = 7) were mixed at 25 °C for 240 min, to determine the adsorption capacity and removal rate. Then the effects of adsorbent dosage (2–25 mg), pH value (3–9), and temperature (25–45 °C) on adsorption performance were also studied under the same methods. For the adsorption isotherm studies, 10 mg of modified biochar was mixed with 25 mL of MB solution (10–200 mg L^−1^, pH = 7) for 240 min. For the kinetics studies, batch experiments were conducted by placing 10 mg of modified biochar into 25 mL of MB solution (100 mg L^−1^ and pH = 7) at different times (2–240 min).

The adsorption effect is the adsorption amount (*q*_e_) and removal rate (*E*) were calculated using eqn (1) and (2).1
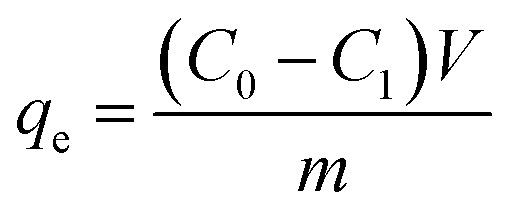
2
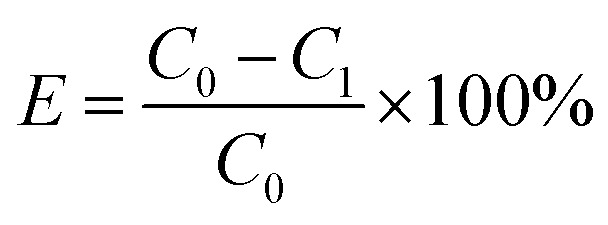
where, *q*_e_ (mg g^−1^) is the equilibrium adsorption amount of the modified biochar to methylene blue; *C*_0_ (mg L^−1^) is the initial concentration of MB; *C*_1_ (mg L^−1^) is the concentration of MB after the adsorption equilibrium; *V* (mL) is the volume of the solution; *m* (g) is the mass of the biochar; *E* (%) is the removal rate.

## 3. Characterization of biochars

**Fig. 1:**
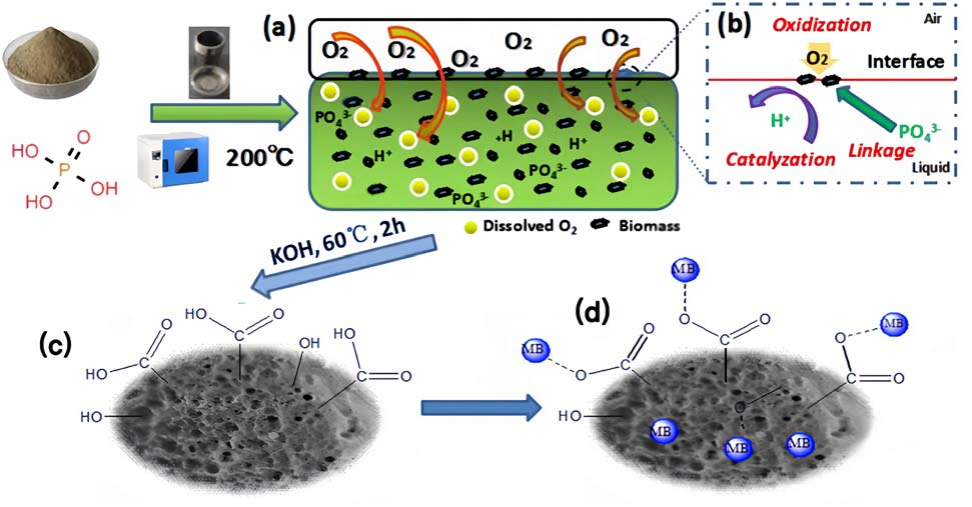
The wet pyrolysis process for the preparation of modified biochar under phosphoric acid and dye adsorption on as-prepared biochar adsorbent: (a) specific reaction during both carbonization and activation, (b) reaction occurred as the interface between liquid and gas, (c) functionalization using KOH, and (d) dye adsorption of MB on.

The schematic workflow of preparing biochar by wet pyrolysis of phosphoric acid is shown in Fig. 1. The pulverized seaweed, coconut shell, or coconut coat powder was mixed with 75% phosphoric acid and heated to 200 °C. Because the decomposition point of H_3_PO_4_ was higher than the temperature of pyrolysis process, the liquid state can be readily maintained at 200. The conversion reaction can be promoted by dissolving oxygen into the reaction system through the creation of a perfect liquid/gas interface in the exposed system.

The wet pyrolysis process for the preparation of modified biochar was summarized in Fig. 1(a). Typically, the degradation and decomposition of cellulose, hemicellulose, and lignin in biomass would occur under the catalysis of protons released by phosphoric acid during the increase of temperature. Residual oxygen participates in the surface oxidation process of biochar, which better increases the functional groups on the biochar surface (Fig. 1(b)). The existence of a proton catalyst, the participation of residual oxygen, the proper selection of reaction temperature, and the utilization of a sealed reactor can facilitate both the simultaneous transformation process of biomass and the surface modification reaction of biochar.

There are two purposes for the mixing of synthetic biochar with KOH. First, the residual phosphoric acid was removed to neutralize the prepared biochar system. Second, the strong alkalinity of potassium hydroxide could effectively destroy the hydrogen bonds between molecules. The hydrogen atoms of –COOH, –OH, –POOH were replaced by potassium (Fig. 1(c)), which is conducive to the adsorption of methylene blue by the modified biochar through ion exchange, formation of hydrogen bonds, *etc.* as illustrated in Fig. 1(d).

### 3.1 SEM-EDS analysis

**Fig. 2:**
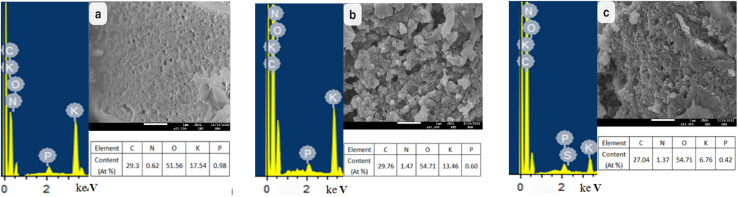
SEM images and EDS data of modified biochars ((a) SEM-EDS of ABC, (b) SEM-EDS of CSBC, (c) SEM-EDS of CCBC).

SEM-EDS of ABC, CSBC, and CCBC were shown in Fig. 2. SEM-EDS diagram (2a) showed that ABC was sponge-like in appearance, with nano-scale pores on its surface. The contents of C, O, and P on the consistency of ABC were equivalent to those of the other two samples, while the range of K was higher than that of the different two samples, while the range of N was slightly lower. SEM-EDS diagrams Fig. 2(b and c) showed that the surface of CSBC and CCBC materials was rough and uneven, showing a stacked shape. The high O/C ratio and the existence of P on the surface of the three biochar samples indicated that large oxygen-containing functional groups and a certain amount of phosphorus-containing functional groups on the surface of biochar, which provided more active sites for the adsorption of pollutants.

### 3.2 BET analysis

**Fig. 3:**
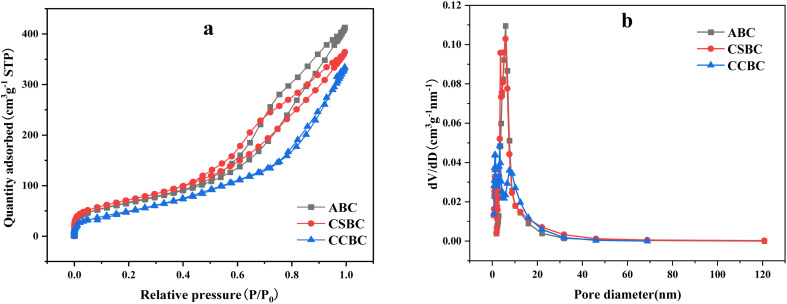
N_2_ adsorption–desorption analysis isotherm and pore size distribution of modified biochars: ((a) N_2_ adsorption–desorption analysis isotherm and (b) pore size distribution).

The characterizations of nitrogen adsorption–desorption and pore size distribution of ABC, CSBC, and CCBC were shown in Fig. 3. The corresponding information of the specific surface area, average pore size, and pore volume was shown in Table 1. The data showed that abundant mesopores were on the surface of ABC, CSBC, and CCBC. The specific surface area of samples was higher than that of biochar prepared by a hydrothermal method.

**Table 1:** BET data of biochar samples

Sample	Specific surface area (m^2^ g^−1^)	Average pore size (nm)	Total pore volume (cm^3^ g^−1^)
ABC	239.80	6.92	0.642
CSBC	259.77	6.04	0.568
CCBC	190.74	7.23	0.508

The modified biochars can be assigned to the type II adsorption isotherm with a classical e H3 hysteresis loop (see Fig. 3), which indicated that the holes on the material surface included a flat slit structure, crack, and wedge structure. In Table 1, the pore size of the modified biochar was mainly ranged from 1 to 20 nm, and the average pore size was 6.92 nm (ABC), 6.04 nm (CSBC), and 7.23 nm (CCBC). The CSBC sample has the highest specific surface area up to 259.77 m^2^ g^−1^. ABS sample showed a slightly lower surface area value. But CCBC sample has the lowest surface area of 190.74 m^2^ g ^−1^.

### 3.3 FT-IR analysis

**Fig. 4:**
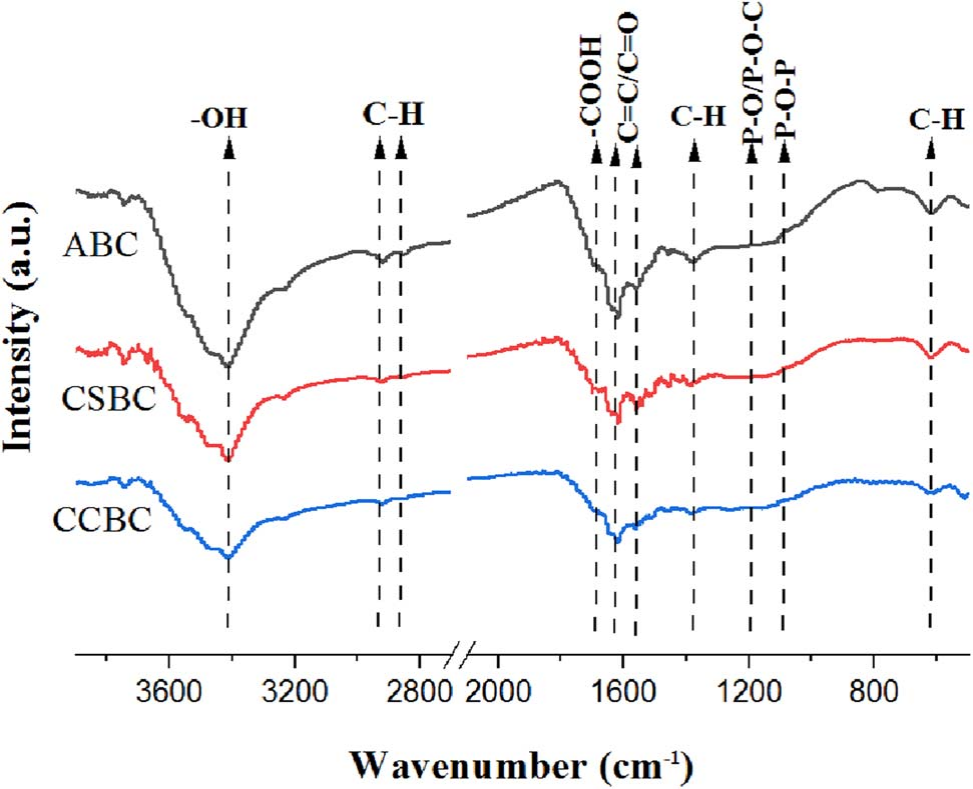
FT-IR spectra of ABC, CSBC, and CCBC.

The functional groups of ABC, CSBC, and CCBC determined through FT-IR were summarized in Fig. 4. For all samples, peaks that belonged to distinct functional groups of all modified biochars were founded, including a strong broad peak of OH at 3440 cm^−1^,^16,17^ a vibration peak of –COOH at 1680 cm^−1^, absorption peaks at 1635 cm^−1^ and 1580 cm^−1^ derived from C covalent bond in the aromatic fragment or C=O in carboxyl structure,^18^ a stretching vibration peak of C–H at 1381 cm^−1^,^19^ P-related functional groups (P=O, P–O–C, P=OOH, and P + –O–) appeared at 1180 cm^−1^ and 1090 cm^−1^.^18,20^ These FT-IR spectra showed that abundant oxygen-containing functional groups and phosphorus-containing functional groups were presented in the biochar samples.

**Fig. 5:**
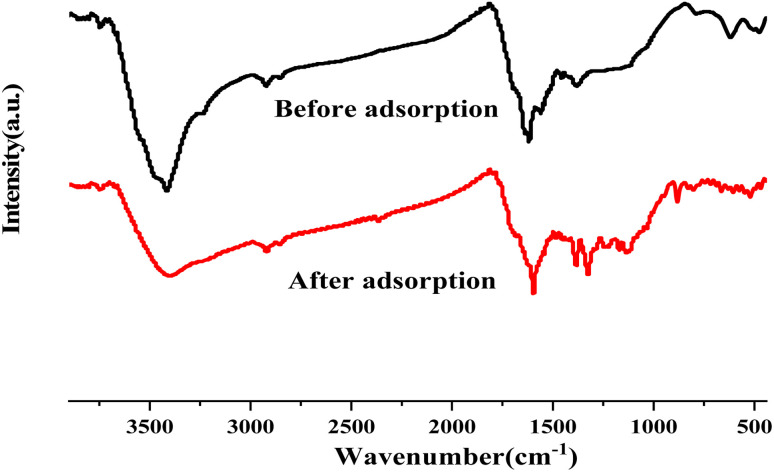
FT-IR spectra of ABC before and after adsorption of methylene blue.

The infrared spectra of seaweed biochar before and after adsorption of methylene blue are characterized. The results are shown in Fig. 5. After adsorption, it is obvious that some infrared peaks have changed in displacement, and the stretching vibration peak of O–H has shifted from 3440 cm^−1^ to 3408 cm^−1^, and the stretching vibration peak of –COOH has shifted from 1680 cm^−1^ to 1660 cm^−1^. This shows that methylene blue has interacted with O–H, –COOH and other groups on the surface of biochar during adsorption, and the interaction mode may be hydrogen bonding, and oxygen-containing groups play an important role in adsorption.

### 3.4 Effect of adsorbent dosage of MB adsorption onto modified biochars

**Fig. 6:**
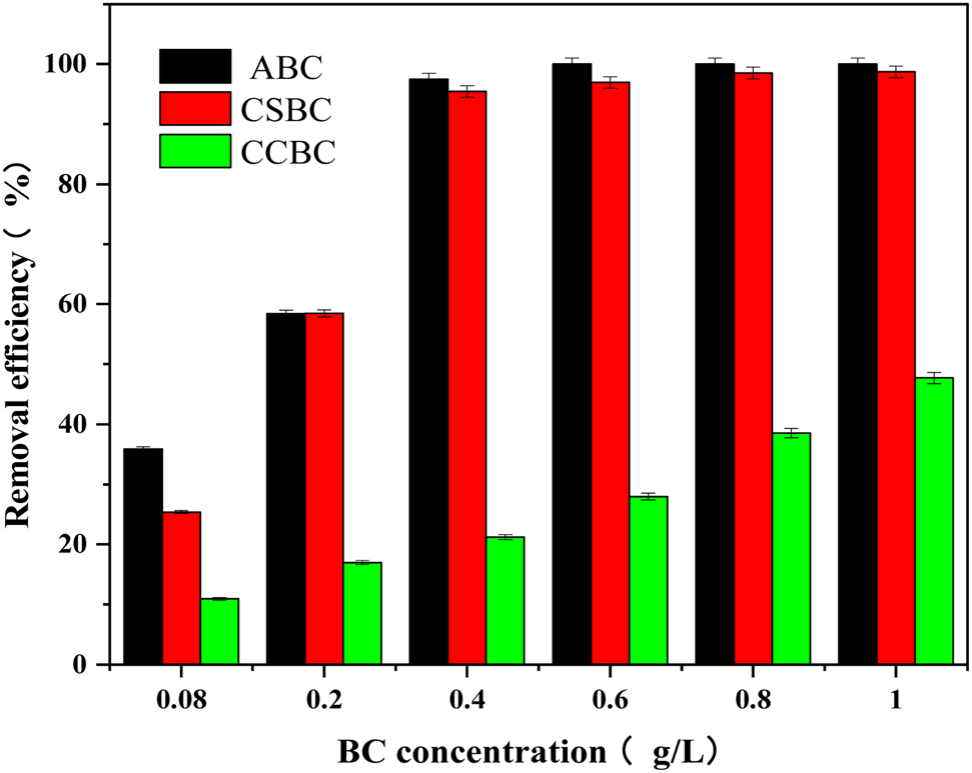
Effect of addition amount on adsorption effect.

The ABC, CSBC, and CCBC with various dosages (2 mg, 5 mg, 10 mg, 15 mg, 20 mg, and 25 mg) were used for the adsorption of MB in water, and the initial concentration of MB was 100 mg L^−1^. Fig. 6 indicated the adsorption effects of three kinds of phosphoric acid-modified biochars on MB were correlated with the additional amount, and ABC and CSBC showed much higher MB adsorption capacities than the CCBC. The removal rates of MB increased from 36.0, 25.4, and 10.9 to 97.5%, 95.4, and 47.7% as the dose of ABC, CSBC, and CCBC increased from 2 to 10 mg. When the additional amount of ABC and CSBC was raised from 2 mg to 10 mg and CCBC was raised from 2 mg to 25 mg, the adsorption effects on MB were linearly correlated with the additional amount. After the dosage of ABC and CSBC was added to more than 10 mg, the adsorption effects of MB were not changed obviously.

### 3.5 Effect of initial pH on MB adsorption onto modified biochars

**Fig. 7:**
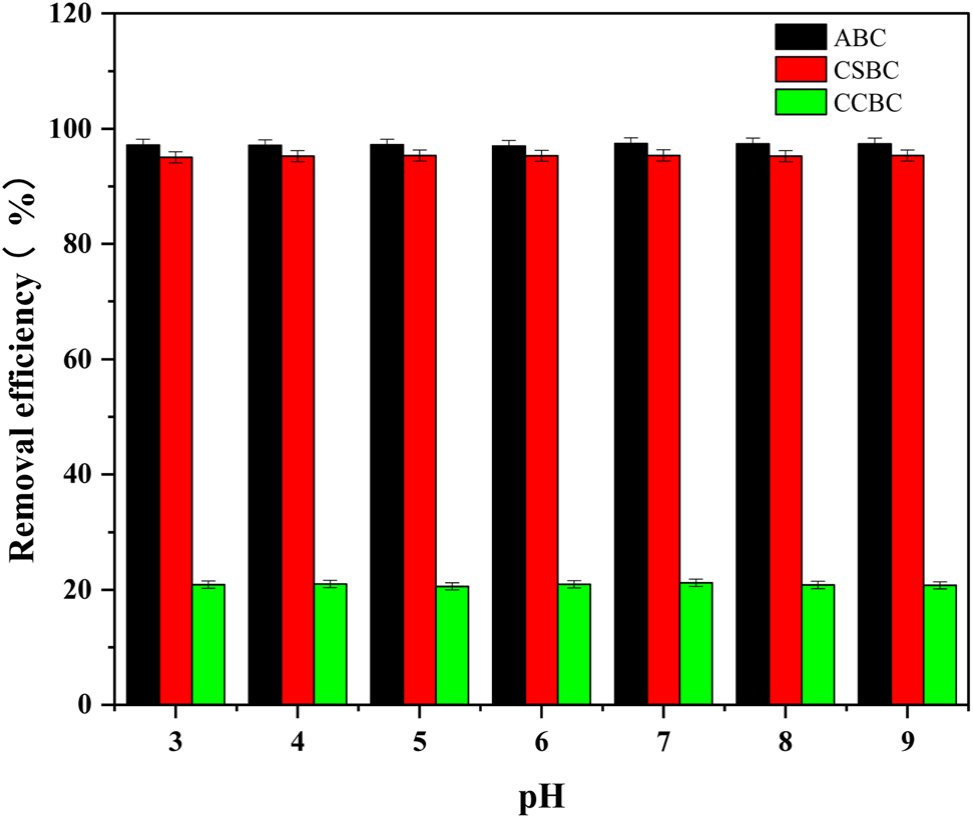
Effect of pH on adsorption effect.

The effects of pH (3–9) in the initial solution on MB adsorption were evaluated (Fig. 7) using 10 mg of modified biochars and 100 mg L^−1^ of MB. Interestingly, the removal rate of MB dye and adsorption capacity of ABC, CSBC, and CCBC remained constant values when the pH value was changed from 3 to 9. The pHpzc of ABC, CSBC, and CCBC were 7.45, 7.53, and 7.59 respectively, which indicated that modified biochars would be electric neutral at pH 3–9. The electrostatic attraction between electrically neutral biochars modified by wet pyrolysis using phosphoric acid and positively charged MB had no significant effect on adsorption. Therefore, in addition to other chemical and physical adsorption effects, we can conclude that electrostatic adsorption can be considered as an influencing factor. Indeed, Faria *et al.* reported that high adsorption ability of MB using modified biochars was attributed to the specific dispersive interactions between dye molecules and activated carbons.

### 3.6 Effect of ambient temperature on MB adsorption onto modified biochars

**Fig. 8:**
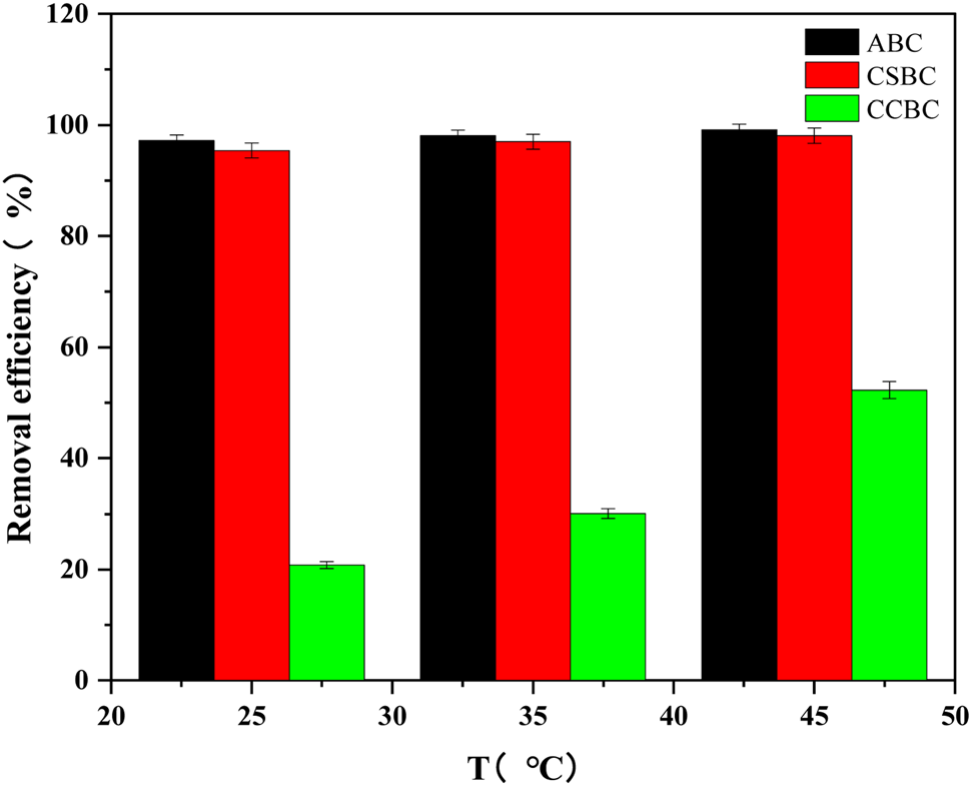
Effect of ambient temperature on adsorption effect.

The effects of ambient temperature on MB adsorption were evaluated (Fig. 8) using 100 mg L^−1^ of MB and 10 mg of modified biochar. With the increase of ambient temperature from 25 °C to 45 °C, the removal efficiency of MB by ABC, CSBC, and CCBC reached the maximum. The increased ambient temperature was conducive to the adsorption and desorption process. The experimental results indicated that the ambient temperature was more conducive to the adsorption process. Moreover, these results confirmed the existence of a large number of oxygen-containing functional groups and the presence of rich mesoporous structures.

### 3.7 Adsorption isotherm and kinetics

**Fig. 9:**
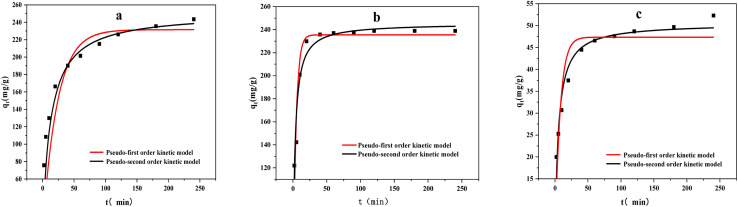
Kinetic fitting curves of adsorption of methylene blue by three kinds of biochar ((a) ABC, (b) CSBC, and (c) CCBC).

The adsorption dynamics behavior was evaluated by the pseudo-first-order and pseudo-second-order models (Fig. 9) .These models are defined by eqn (3) and (4):

3 *q*_*t*_ = *q*_e_(1 − e^−*k*_1_*t*^)

4 *q*_*t*_ = *k*_2_*q*_e_^2^*t*/(1 + *k*_2_*q*_e_*t*)

where, *q*_e_ (mg g^−1^) is the equilibrium adsorption amount of the modified biochar to methylene blue; *q*_*t*_ (mg g^−1^) is the adsorption amount of the modified biochar to methylene blue at time (*t*); *k*_1_ (min^−1^) and *k*_2_ (g mg^−1^ min^−1^)are the pseudo-first-order and pseudo-second-order models kinetics constants, respectively.

**Fig. 10:**
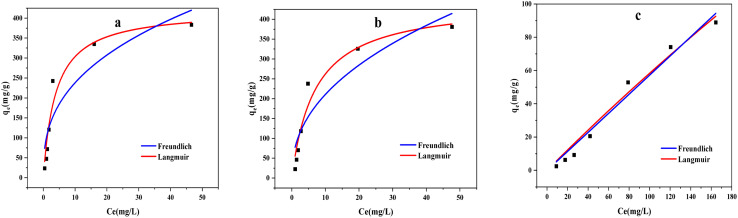
Equilibrium adsorption isotherms of methylene blue by modified biochars ((a) ABC, (b) CSBC, and (c) CCBC).

The Langmuir model and Freundlich model (Fig. 10) were also employed to analyze the adsorption processes. These models are defined by eqn (5) and (6):

5 *q*_e_ = (*q*_max_*K*_L_*C*_e_)/(1 + *K*_L_*C*_e_)

6 *q*_e_ = *K*_F_*C*_e_^1/*n*^

where, *C*_e_ (mg L^−1^) is the mass concentration of methylene blue during the adsorption equilibrium of the modified biochar; *q*_max_ (mg g^−1^) is theoretical maximum adsorption of methylene blue from modified biochar; *K*_L_ is the Langmuir constant, *K*_F_ is the Freundlich coefficient.

As summarized in Fig. 9 and Table 2, the regression coefficient values of all the adsorption behavior derived from three modified biochars would be suitable for the pseudo-second-order model. The theoretical *q*_e_ values could be calculated based on the equilibrium concentrations of MB on the adsorbed ABC, CSBC, and CCBC. Thereby, the removal efficiencies of MB dye can reach up to 97.5%, 95.4%, and 21.3%, respectively. The calculated *q*_e_ values were consistent with the theoretical values. Furthermore, the fitting plots showed a great linearity with *R*^2^ values > 0.90. Accordingly, the adsorption kinetics can be considered to follow a pseudo-second-order model, inferring the mechanism of a chemisorption process for the MB dye removal.

**Table 2:** Fitting parameters of the adsorption kinetics models of three kinds of biochar

Samples	Pseudo-first-order model			Pseudo-second-order model		
	*q*_e_ (mg g^−1^)	*k*_1_ (min^−1^)	*R* ^2^	*q*_e_ (mg g^−1^)	*k*_2_ (g (mg^−1^ min^−1^))	*R*2
ABC	231.5	0.043	0.992	251.1	3.13 × 10^−4^	0.998
CSBC	235.5	0.233	0.899	245.4	0.002	0.952
CCBC	47.4	0.13	0.840	50.5	0.004	0.951

Moreover, both Langmuir model and Freundlich model were utilized to describe the ideal monolayer adsorption and the nonideal multilayer adsorption. In Fig. 10 and Table 3, Langmuir would be a better model to fit the adsorption behavior of MB by the ABC, CSBC, and CCBC absorbent than that of the Freundlich isotherm based on the regression coefficients. Therefore, we presumed that the adsorption process might involve with a monolayer adsorption process, and the adsorption of MB on the ABC, CSBC, and CCBC seemed to be a spontaneous process.

**Table 3:** Parameters of adsorption isotherm model for modified biochars

Samples	Langmuir			Freundlich		
	*q*_m_ (mg g^−1^)	*k*_L_ (L^−1^ mg^−1^)	*R* ^2^	*k*_F_ (mg^−1^ g^−1^)	*n*_F_	*R* ^2^
ABC	108.2	0.257	0.953	101.9	0.369	0.852
CSBC	63.7	0.143	0.953	77.6	0.434	0.869
CCBC	0.6	5.939 × 10^−4^	0.977	0.6	1.01	0.976

## 4. Conclusion

The present study showed that three viable novel adsorbents ABC, CSBC, and CCBC could be prepared by wet pyrolysis from seaweed, coconut shell, and coconut coat through phosphoric acid activation. Using phosphoric acid as a solvent, the wet pyrolysis was conducted under normal pressure, with simple operation, low energy consumption, and excellent performance for cation adsorption. This study compares the KOH modified biochar,^21^ phosphoric acid modification of method is more convenient. Seaweed, coconut shell, and coconut coat were all considered as excellent agro-waste, which were abundantly disposed in Hainan province without any treatment. To the best of our knowledge, phosphoric acid and biomass type were important parameters in structure control regarding micromesoporosity and surface chemistry. The adsorption processes of MB at room temperature were suitably fitted with Langmuir models and the quasi-second-order adsorption kinetics model, which showed that MB adsorbed in modified samples ABC and CSBC were higher than those of carbons prepared by similar methods. These results were also consistent with the value of BET surface area and the amount of rich oxygen-containing functional groups present on these carbons. MB adsorbed onto the modified carbons with supermicropores might mainly regulate by both the dispersive interaction and the electrostatic interaction. Furthermore, the presence of oxygen-containing functional groups not only promoted the electrostatic interactions, but also reduced the dispersive interactions. The full characterizations on these carbons proved that seaweed and coconut shell might be greatly renewable materials for the preparation of promising activated carbons for pollution remediation and other related fields.

## Conflicts of interest

There are no conflicts to declare.

## Supplementary Material
